# Robust Association between Acute Kidney Injury after Radical Nephrectomy and Long-term Renal Function

**DOI:** 10.3390/jcm9030619

**Published:** 2020-02-25

**Authors:** Won Ho Kim, Kyung Won Shin, Sang-Hwan Ji, Young-Eun Jang, Ji-Hyun Lee, Chang Wook Jeong, Cheol Kwak, Young-Jin Lim

**Affiliations:** 1 Department of Anesthesiology and Pain Medicine, Seoul National University Hospital, Seoul National University College of Medicine, Seoul 03080, Korea; skwskw1@naver.com (K.W.S.); taepoongshin@gmail.com (S.-H.J.); na0ag2@hotmail.com (Y.-E.J.); muslab@hanmail.net (J.-H.L.); limyjin@snu.ac.kr (Y.-J.L.); 2 Department of Urology, Seoul National University Hospital, Seoul National University College of Medicine, Seoul, 03080, Korea; drboss@korea.com (C.W.J.); mdrafael@snu.ac.kr (C.K.)

**Keywords:** acute kidney injury, radical nephrectomy, chronic kidney disease, functional change ratio

## Abstract

The association between acute kidney injury (AKI) and long-term renal function after radical nephrectomy has not been evaluated fully. We reviewed 558 cases of radical nephrectomy. Postoperative AKI was defined by the Kidney Disease: Improving Global Outcomes (KDIGO) serum creatinine criteria. Values of estimated glomerular filtration rate (eGFR) were collected up to 36 months (median 35 months) after surgery. The primary outcome was new-onset chronic kidney disease (CKD) stage 3a or higher or all-cause mortality within three years after nephrectomy. The functional change ratio (FCR) of eGFR was defined as the ratio of the most recent GFR (24–36 months after surgery) to the new baseline during 3–12 months. A multivariable Cox proportional hazard regression analysis for new-onset CKD and a multivariable linear regression analysis for FCR were performed to evaluate the association between AKI and long-term renal outcomes. A correlation analysis was performed with the serum creatinine ratio and used to determine AKI and FCR. AKI occurred in 43.2% (*n* = 241/558) and our primary outcome developed in 40.5% (*n* = 226/558) of patients. The incidence of new-onset CKD was significantly higher in patients with AKI than those without at all follow-up time points after surgery. The Cox regression analysis showed a graded association between AKI and our primary outcome (AKI stage 1: Hazard ratio 1.71, 95% confidence interval 1.25–2.32; AKI stage 2 or 3: Hazard ratio 2.72, 95% confidence interval 1.78–4.10). The linear regression analysis for FCR showed that AKI was significantly associated with FCR (β = −0.168 ± 0.322, *p* = 0.011). There was a significant negative correlation between the serum creatinine ratio and FCR. In conclusion, our analysis demonstrated a robust and graded association between AKI after radical nephrectomy and long-term renal functional deterioration.

## 1. Introduction

Acute kidney injury (AKI) is a frequent complication after radical nephrectomy, with an incidence of up to 53.9% [1,2,3], and is associated with the development of chronic kidney disease (CKD) [4]. Although there are a few studies regarding this association after radical nephrectomy, the associations of AKI with new-onset CKD or long-term renal function after radical nephrectomy have not been evaluated fully [1,2,3]. To suggest a causal relationship, a dose-response relationship between AKI stages and CKD should be demonstrated [5]. However, the graded association of AKI stages with long-term renal function or the linear association of the ratio of creatinine used to diagnose AKI with a functional change ratio has not been evaluated before.

Several studies evaluated the association between AKI and new-onset CKD after radical nephrectomy. A previous retrospective study showed a significant association between AKI and new-onset CKD [2]. In this study, the new-onset CKD was defined as a 40% or more decrease in estimated glomerular filtration rate (eGFR) from the preoperative baseline. However, this study reviewed only a small number of 106 patients over a long period of eighteen years. Furthermore, there were 26% of patients with a baseline eGFR < 60 mL/min/1.73 m^2^. These patients should have been excluded from their analysis. Another retrospective study reported AKI after radical nephrectomy is a risk factor for new-onset CKD [1]. This study defined CKD as eGFR < 60 mL/min/1.73 m^2^, but 18% of patients with baseline eGFR < 60 mL/min/1.73 m^2^ were included in the analysis. These two studies performed a logistic regression analysis to evaluate whether AKI was an independent risk factor of new-onset CKD. However, new-onset CKD is a time-to-event outcome and logistic regression analysis is not an adequate analysis for the outcome, hence both the Cox regression analysis and Kaplan–Meier survival analysis are required. In addition, mortality is a competing risk with new-onset CKD and mortality should be combined with the new-onset CKD for the Cox regression analysis.

As such, although a few previous studies have reported the association between AKI after nephrectomy and the risk of development of postoperative CKD, most studies analyzed a relatively small number of patients with different outcome definitions and used inadequate statistical analyses for the time-to-event outcome. As the appropriate statistical analyses were not selected, the linear relationship of the AKI stages with long-term renal functional change [6] or the graded association of AKI stages with CKD have not been evaluated. Therefore, we conducted a retrospective study evaluating the graded association of AKI stages with new-onset CKD and the linear association of AKI stages with renal functional change ratio from new-baseline renal function after radical nephrectomy to assess whether the association of AKI and CKD after radical nephrectomy is robust and has a dose-response relationship. We excluded the patient who received donor nephrectomy for kidney transplantation from our analysis as these patients have different renal functional profiles after surgery. This difference is attributed to the different distribution of patient age and comorbidities [7,8].

## 2. Methods

### 2.1. Patient Selection

This single-center retrospective observational study was approved by the institutional review board (IRB) of Seoul National University Hospital (1907-172-1050). Written informed consent was waived by the IRB due to the retrospective nature of the present study. We reviewed electronic medical records of the patients who were ≥ 20 years old, had a renal mass, and underwent radical nephrectomy, regardless of surgical techniques between 2010 and 2015. Among the 670 patients who underwent radical nephrectomy, the patients with baseline eGFR < 60 mL/min/1.73 m^2^ (*n* = 50), the absence of contralateral kidney (*n* = 9), or missing baseline serum creatinine (*n* = 0) or three or more follow-up loss of serum creatinine or eGFR values among 3, 12, 24, and 36 months after surgery (*n* = 22) were excluded. We also further excluded patients who underwent subsequent nephrectomy *(n* = 2), catheter ablation of remaining kidney (*n* = 5), chemotherapy with a nephrotoxic agent (*n* = 4), or received other nephrotoxic drugs including diuretics (*n* = 3), nephrotoxic antibiotics (*n* = 4), and radiocontrast media (*n* = 13) during our follow-up period because these could affect residual renal function. The remaining 558 patients were included in the final analysis.

### 2.2. Patient Data and Outcome Measurements

Demographic, baseline characteristics, and surgery-related data which were known to be associated with renal function after nephrectomy were obtained from our electronic medical records (Table 1) [1,2,3,9]. Serum creatinine values measured at 3, 12, 24, 36 months after surgery were collected. GFR values were estimated at these time points after surgery by the Modification in Diet and Renal Disease (MDRD) study equation [10]. Decisions regarding radical nephrectomy and the type of surgical approach were made by surgeons based on tumor characteristics.

The primary outcome was new-onset CKD or higher or all-cause mortality within three years after radical nephrectomy. The new-onset CKD was defined as a decrease in eGFR < 60 mL/min/1.73 m^2^ or the initiation of chronic hemodialysis [11]. The decrease in eGFR should be identified by at least two consecutive measurements separated by an interval of at least three months [12]. The primary outcome variable was defined as a time-to-event outcome.

Secondary outcomes included the long-term functional change ratio (FCR) of GFR, which was defined as the most recent GFR/new baseline GFR after surgery [6]. New baseline GFR was defined as the latest value available during 3–12 months after surgery considering that renal function recovers after the initial drop immediately after surgery. The most recent GFR value of at least 24 months after surgery was compared to this new baseline.

Postoperative AKI was defined by the creatinine criteria of Kidney Disease: Improving Global Outcomes (KDIGO), which was determined according to the maximal change in serum creatinine levels during the first seven postoperative days (stage 1: 1.5–1.9; stage 2: 2–2.9; stage 3: More than 3-fold increase of baseline, respectively, also stage 1: when serum creatinine increased by 0.3 mg/dL within 48 h) [13,14]. The most recent serum creatinine level measured before surgery was used as the baseline.

### 2.3. Statistical Methods

SPSS software version 25.0 (IBM Corp., Armonk, NY, USA) and STATA/MP version 15.1 (StataCorp, College Station, TX, USA) were used to analyze the data. The visual inspection of histograms and quintile–quintile plots was performed to determine the normality of the data. Continuous variables were presented as mean ± SD for normally distributed data or median (25th, 75th percentiles) for non-normally distributed data. For all analyses, *p* < 0.05 was considered statistically significant. Baseline characteristics and surgery-related parameters had missing values in <1% and these missing were considered to be due to missing completely at random. The incidences of missing in the baseline parameters were reported and they were not replaced. Missing values of serum creatinine and eGFR at 3, 12, 24, 36 months were reported and were not replaced, and an analysis was performed with available data. One of our investigators (W.K.) verified artifact or incorrect values according to our source document and excluded them from our analysis. The following are a summary of our main analyses. Firstly, the serial eGFR values up to 36 months after surgery and cumulative incidences of postoperative development of new-onset CKD were depicted and compared at all follow-up time points between the patients without AKI, those with AKI stage 1, and those with AKI stage 2 or 3. The absolute eGFR values were compared among no-AKI, AKI stage 1, and stage 2 or 3 at each time point by one-way analysis of variance. The cumulative incidence of new-onset CKD was compared between the same groups by the chi-square test. Bonferroni correction was used to adjust for the increased type 1 error by multiple comparisons (*p* < 0.05/4 = 0.012).

Secondly, we scrutinized the relationship between postoperative AKI and the development of new-onset CKD during the 60 months after surgery through a multivariable Cox proportional hazard regression analysis. Proportional hazard assumptions were tested by visual inspection of log-minus-log survival plots for categorical variables and restricted cubic splines for continuous variables [15,16]. Before conducting multivariable analysis, multicollinearity among covariates was evaluated using the variance inflation factor. Variables with variance inflation factor > 5 were excluded from the analysis. Cases with missing values of the covariates were excluded from the Cox regression analysis for the complete case analysis. All collected perioperative variables listed in Table 1 were entered into the multivariable model to adjust for the association between AKI and CKD. AKI as a binary variable or the stages of AKI an ordinal variable were entered alternatively as a covariate in the regression model. All covariates entered the model without stepwise variable selection or a univariable screening. Calibration of the Cox regression model was evaluated by Gronnesby and Borgan test [17] and discrimination of the model was measured by Harrell’s *c* and Somers’ *D* [18].

Thirdly, a Kaplan–Meier survival curve analysis of our primary outcome was performed among the different groups of the patients without AKI, those with AKI stage 1, and those with AKI stage 2 or 3. The log-rank test was used to determine statistical significance between groups.

Fourthly, as analysis for the secondary outcome of FCR, we performed a multivariable linear regression analysis to elucidate whether AKI is significantly associated with the long-term FCR of the most recent eGFR to a new postoperative renal functional baseline. Linear regression model assumptions were evaluated using residual plots and scatter plots of our data. Neither stepwise variable selection nor univariable screening was performed. Multicollinearity among covariates was evaluated using the variance inflation factor.

Fifthly, a scatter plot was depicted relating the distribution of FCR across the serum creatinine ratio used to determine AKI. We tested whether the distribution of FCR is different among the different stages of AKI by univariable Spearman correlation analysis.

Sixthly, as a post-hoc analysis to evaluate the postoperative renal function in terms of KDIGO CKD stages [12], the distribution of KDIGO CKD stages during the follow-up period was compared between those with and without AKI.

Finally, as a post-hoc analysis to evaluate the association of the type of surgical procedure and AKI, we compared the incidence of AKI between open surgery and laparoscopic or robotic surgery. In addition, to evaluate the association between the surgical procedure and our primary outcome, we performed Kaplan–Meier survival curve analysis. The log-rank test was used to determine statistical significance.

## 3. Results

Demographics and perioperative parameters were shown in Table 1. The incidence of AKI was 43.2% (*n* = 241/558) (stage 1: *n* = 160 (28.7%); stage 2 or 3: *n* = 81 (14.5%)). Our primary outcome developed in 40.5% (*n* = 226/558) of patients. The median follow-up of renal function was 35 (25–37) months. The numbers of patients lost to follow-up at 2 and 3 years after surgery were 143 (24.7%) and 305 (52.8%) (Table S1).

Table 1 shows the baseline characteristics of the patients. Median patient age was 60 years and 60.7% of patients were male. Median FCR at three years after radical nephrectomy was 0.94 in patients without AKI and 0.80/0.72 for patients with stage 1/2 or 3 AKI.

**Figure 1:**
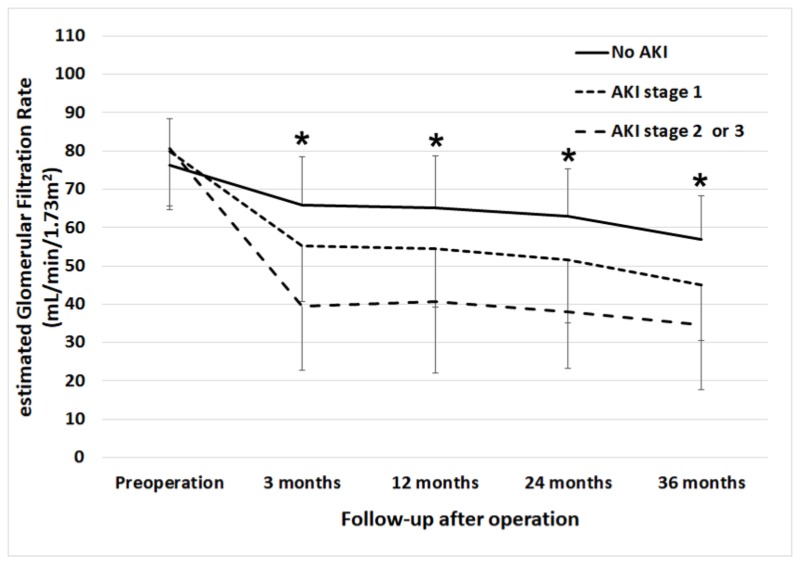
Serial change in mean estimated glomerular filtration rate before surgery to three years after surgery according to the stages of acute kidney injury. * Significant difference between groups.

**Figure 2:**
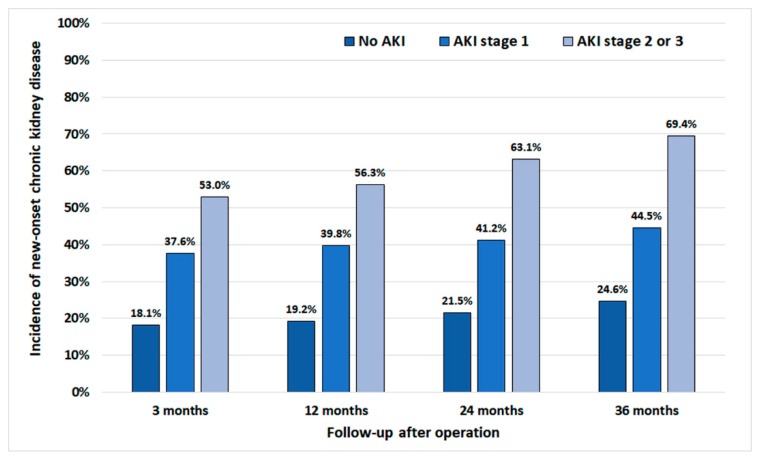
Comparison of the cumulative incidence of chronic kidney disease (estimated glomerular filtration rate (eGFR) <60 mL/min/1.73 m^2^) at follow-up time points between the patients without acute kidney injury (AKI), those with stage 1 AKI, and those with stage 2 or 3 AKI. There were significant differences in the incidence of chronic kidney disease at all time-points.

Figure 1 shows the comparison of a time-dependent change in eGFR at 3 months, 1, 2, and 3 years after radical nephrectomy among the patients without AKI, those with stage 1 AKI and those with stage 2 or 3 AKI. There were significant differences in eGFR values between groups in all time points (*p* < 0.001). Figure 2 shows comparisons of the cumulative incidence of new-onset CKD at the time points of follow-up between those with and without AKI. While 24.7% of patients with no history of AKI developed CKD until three years after surgery, 69.5% of patients developed CKD with a history of stage 2 or 3 AKI (*p* < 0.001). There were significant differences in the incidence of new-onset CKD at all time points (*p* < 0.001).

Table 2 shows the results of the multivariable Cox regression analysis to predict the development of new-onset CKD during the three years after radical nephrectomy. All covariates met the proportional hazard assumptions. Postoperative AKI was identified as an independent risk factor for the development of new-onset CKD (multivariable-adjusted hazard ratio (HR) 2.46, 95% confidence interval (CI) 1.70–3.63, *p <* 0.001). There was a graded association with CKD for stage 1 (HR 1.71, 95% CI 1.25-2.32, *p <* 0.001) and stage 2 or 3 AKI (HR 2.72, 95% CI 1.78–4.10, *p* < 0.001). The performance of our multivariable Cox model in terms of Harrell’s *c* and Somers’ *D* was 0.67 and 0.60, respectively. Our Cox regression model showed good calibration (Gronnesby and Borgan test: χ^2^ = 0.876, *p* = 0.441).

**Figure 3:**
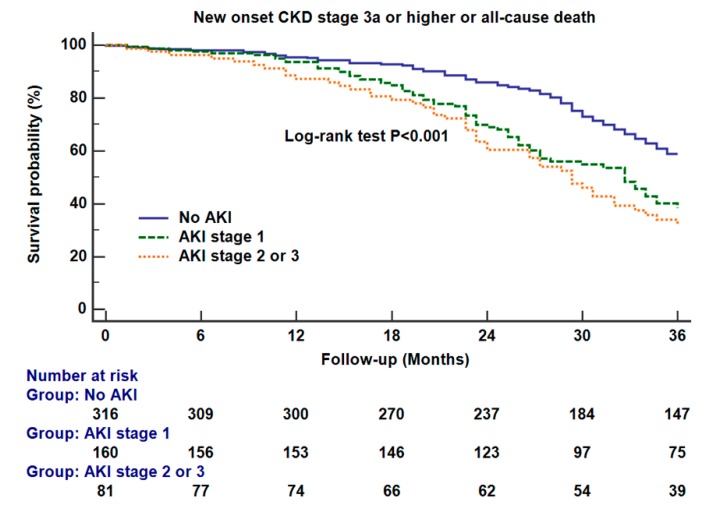
Kaplan–Meier survival curve analysis for new-onset chronic kidney disease stage 3a or higher (estimated glomerular filtration rate < 60 mL/min/1.73 m^2^) or all-cause mortality as the primary outcome between different AKI groups. There were significant differences between the no AKI and AKI stage 1 groups (log-rank test *p* < 0.001) and between AKI stage 1 and stage 2 or 3 (log-rank test *p* = 0.023). The numbers of patients who had follow-up eGFR values at each time point were shown at the bottom of the figure.

Figure 3 shows Kaplan–Meier survival curve analysis for our primary outcome between different AKI groups. There were significant differences between the no AKI and AKI stage 1 groups (log-rank test *p* < 0.001) and between AKI stage 1 and stage 2 or 3 (log-rank test *p* = 0.023). The numbers of patients who had follow-up eGFR values at each time point were shown in Figure 3**.**

Table 3 shows the results of the multivariable linear regression analysis for FCR after radical nephrectomy. All covariates met the linear assumptions. Postoperative AKI was independently associated with the FCR of the most recent follow-up (β = −0.168 ± 0.322, *p* = 0.011). The performance of our multivariable prediction in terms of R^2^ was 0.28 and there was no significant multicollinearity between covariates.

**Figure 4:**
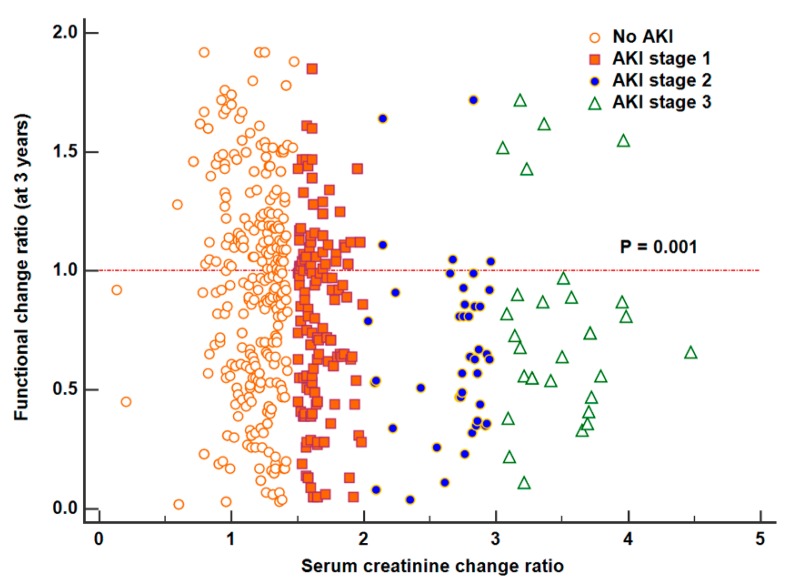
Distribution of the long-term functional change ratio across the different creatinine ratios which were used to determine acute kidney injury stages. There was a significant correlation between the functional change ratio and creatinine change ratio from baseline (*p* < 0.001).

Figure 4 shows the distribution of FCR across the serum creatinine ratio used to determine AKI and AKI stages. There was a significant negative correlation between the FCR and the serum creatinine ratio (*p* < 0.001). This means the residual renal function deteriorates more as the stages of AKI increase.

**Figure 5:**
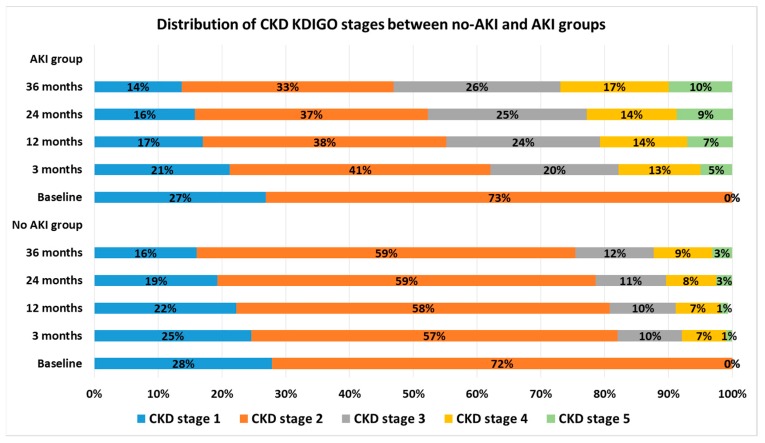
Comparison of the distribution of the Kidney Disease: Improving Global Outcomes chronic kidney disease (KDIGO CKD) stages during the follow-up period of 36 months between the patients with and without AKI after radical nephrectomy.

Figure 5 compares the distribution of the KDIGO CKD stages during our follow-up period of 36 months between those with and without AKI. There was a significant difference in the distribution of CKD stages between the patients with and without AKI.

The incidence of postoperative AKI was significantly lower in the laparoscopic or robotic surgery group (*n* = 77, 32.6%) compared to the open surgery group (*n* = 169, 49.4%, *p* < 0.001) (Table S2). However, there was no significant difference in the renal survival between the laparoscopic or robotic group and the open group (Log-rank test *p* = 0.865) (Figure S1). As a supplementary analysis, the incidence and type of postoperative complication were compared between the patients with and without AKI (Table S3). There was no significant difference between groups.

## 4. Discussion

We evaluated the association between AKI after radical nephrectomy and long-term renal function through a multivariable Cox regression analysis and other sensitivity analyses in a retrospective cohort during a 36-months follow-up. The association between AKI and CKD was revealed again in this specific surgical setting of radical nephrectomy. Furthermore, we demonstrated the graded associations between AKI stages and new-onset CKD as well as between AKI stages and the FCR at 24–36 months after surgery. Our linear regression analysis also showed that AKI was an independent predictor of FCR. Therefore, we added a convincing body of evidence that there is a robust and linear relationship between AKI and long-term renal function after radical nephrectomy. Given the high incidence of new-onset CKD after radical nephrectomy in patients with AKI, clinical trials are urgently required to find a modifiable risk factor to mitigate the risk of AKI.

According to the previous studies comparing radical and partial nephrectomy, radical nephrectomy is more strongly associated with new-onset CKD after surgery [19,20,21,22]. A recent meta-analysis compared the postoperative incidence of CKD stage 3a or higher (eGFR < 60 mL/min/1.73 m^2^) between radical and partial nephrectomy and reported a higher risk of CKD for radical nephrectomy [23]. However, these studies simply compared renal functional outcomes between radical and partial nephrectomy and cannot confirm the causal relationship between radical nephrectomy and new-onset CKD. A recent study constructed a simplified nomogram that predicts postoperative renal functional decline after nephrectomy and could help determine candidates for partial nephrectomy [24]. For stage 1 renal tumors, oncologic outcomes were not different between partial and radical nephrectomy [25,26]. However, partial nephrectomy is associated with a lower incidence of postoperative renal functional decline and lower mortality compared to radical nephrectomy [27,28]. Therefore, nephron-sparing surgery has been proposed as a standard of care for small renal cell carcinomas [29]. However, radical nephrectomy is still an important option when partial nephrectomy is unfeasible [30].

In surgeries other than nephrectomy, AKI has been reported to be closely associated with the development of CKD and increased mortality [4,31]. The association between AKI and CKD was also reported in non-surgical patients [32,33]. However, this association was not found in patients with a history of septic shock during one-year follow-up [34]. Furthermore, there have been only a few studies investigating the association between AKI and long-term renal functional decline for radical nephrectomy. Based on our literature review, there have been three studies that investigated the association between postoperative AKI and the development of progressive chronic kidney disease [1,2,3]. The incidence of AKI was reported to be 34% to 49% and the association between AKI and CKD was strong, with a three to four-fold higher risk pertaining to the odds ratio, although the CKD was defined differently with varying durations of follow-up. The incidence of AKI in our study was higher than in the study of Cho et al., in which it was 33.7% [1], but lower than that of Garfalo et al. at 49.1% [2]. The highest incidence of 53.9% was reported in patients undergoing radical nephrectomy with simultaneous inferior vena cava (IVC) thrombectomy [3]. This high incidence may be due to the frequent implementation of cardiopulmonary bypass and clamping of IVC and the contralateral renal vein. The variance in the incidence of AKI may also be attributed to different diagnostic criteria and the exclusion of urine output criteria. We did not use the urine output criteria because hourly urine output was not accurately recorded in our patients and the oliguria cutoff associated with AKI could be different in the perioperative setting [35,36]. Urine output criteria are regarded as unreliable in predicting AKI in the perioperative environment because oliguria may develop simply due to decreased preload [37], or external obstruction of the urinary tract [38].

Renal function after donor nephrectomy was ascertained in previous studies, which could be compared with our study results [39,40,41]. Compensatory hypertrophy develops in the remaining kidney [39,40,41]. Serum creatinine levels increased to 20% above baseline but usually remained within normal reference range [7,42]. However, in our study, immediate postoperative AKI developed in 43.2% and more than half of our patients with AKI eventually developed CKD of eGFR <60 mL/min/1.73 m^2^ during three years of follow-up. These differences in renal outcomes between kidney donors and our patients with renal cell carcinoma could be attributed to patient characteristics. Our cancer patients are older and have more comorbidities [7,8], which could aggravate the renal functional decline. Our incidence of new-onset CKD during 35 months of median follow-up after radical nephrectomy is similar to previous studies of 38% [1] or 39.6% [2], although the follow-up duration differed. Another previous study reported a higher incidence of 65%, although this study included 26% of patients with pre-existing CKD before surgery [21].

Since AKI after radical nephrectomy is associated with long-term renal functional decline, efforts should be made to reduce the risk of AKI after radical nephrectomy. Creatinine increase after radical nephrectomy would be due to both losses of nephrons in the resected kidney and injury of the contralateral kidney. Although we cannot reduce the loss of nephrons in patients for whom nephron-sparing surgery is not feasible, injury of the contralateral kidney could be prevented by hemodynamic optimization during the perioperative period [43]. Previously reported risk factors of male sex, old age, high body-mass index, hypertension, diabetes mellitus, preoperative proteinuria, high preoperative eGFR are not modifiable [1,2,21,44,45,46]. Comorbidities such as hypertension or diabetes could contribute to the underlying medical renal disease and long-term renal functional deterioration [47]. Our analysis also identified unmodifiable predictors, such as preoperative hypertension, diabetes mellitus, and low baseline eGFR. A previous pilot study reported that remote ischemic conditioning reduced the incidence of AKI in patients undergoing partial nephrectomy [48]. Further studies are required to find modifiable risk factors of AKI or CKD progression after radical nephrectomy [9] and determinants for adaptive hyperfiltration in the contralateral kidney [39].

All surgical procedures including open, laparoscopic, and robotic surgery were pooled in our analysis. A previous study showed that postoperative risk of AKI was halved for the patients treated by robotic or laparoscopic partial nephrectomy when compared with open surgery [49]. To further evaluate this association in our study cohort, we compared the incidence of AKI and the renal survival between the groups of surgical procedure. The incidence of AKI was significantly lower in the laparoscopic or robotic surgery group compared to open surgery. However, there was no significant difference in our primary outcome according to the type of surgical procedure both for Cox regression analysis and survival curve analysis. This may suggest that a surgical procedure may affect short-term outcomes but not long-term renal survival after radical nephrectomy.

Our study should be interpreted under several limitations. Firstly, our study was a retrospective study of a single tertiary care center. Minimally invasive and open nephrectomy were mixed in our population. Although a multivariable analysis and sensitivity analyses were performed, unknown and unmeasured biases could have affected our results. Secondly, there was about 50% follow-up loss of renal function at 36 months after surgery, as shown in Figure 3. A prospective observational study with less loss of follow-up is required for a more valid analysis. Thirdly, our study cohort included patients who might have been considered to undergo partial nephrectomy simply according to the tumor stage provided in Table 1. This may limit the applicability of our data to the current clinical practice. However, other indications of radical nephrectomy such as locally advanced cases, multifocality, and distant metastasis should be considered for this. Fourthly, the exact causes of new-onset CKD during our follow-up period were not elucidated. Although the main cause would be functional declines in the remaining kidney, other pathologies such as new-onset glomerulonephritis could have been included in our primary outcome.

## 5. Conclusions

AKI after radical nephrectomy was associated with long-term renal functional deterioration in a dose-response manner. AKI was an independent risk factor of new-onset CKD in patients without baseline CKD. There was a significant linear relationship between the creatinine ratio used to determine AKI and FCR at 24–36 months after surgery. These associations were stronger in higher stages of AKI. Therefore, there seems to be a robust and strong association between AKI after radical nephrectomy and long-term renal functional decline after surgery. It is required to find modifiable risk factors of AKI and interventions to attenuate AKI to improve long-term renal function after radical nephrectomy.

## Figures and Tables

**Table 1:** Patient characteristics and perioperative parameters according to acute kidney injury (*n =* 558).

Variables	Patients	Proportion without Missing (%)
Demographic data		
Age, year	60 (51–68)	100
Female, *n*	171 (30.6)	100
Body-mass index, kg/m^2^	24.4 (22.6–26.2)	100
Baseline medical status		
Hypertension, *n*	287 (51.4)	100
Diabetes mellitus, *n*	98 (17.6)	100
Cerebrovascular accident, *n*	11 (2.0)	100
Angina pectoris, *n*	9 (1.6)	100
Preoperative hemoglobin, g/dL	13.5 (12.0–14.6)	100
Preoperative serum albumin level, mg/dL	4.3 (4.1–4.6)	99.8
Preoperative proteinuria, *n*	67 (12.0)	100
Preoperative hematuria, *n*	53 (9.5)	100
Preoperative eGFR, mL/min/1.73 m^2^		100
eGFR ≥ 90 mL/min/m^2^	153 (27.4)	
eGFR 60–89 mL/min/1.73m^2^	405 (72.6)	
Surgical parameters		
Surgery type, *n*		100
Laparoscopic	223 (40.0)	
Robot-assisted	9 (1.6)	
Open	325 (58.2)	
Clinical stage, *n*		100
T1a/ T1b	141 (25.3)/152 (27.2)	
T2a/ T2b	154 (27.6)/61 (10.9)	
T3a/T3b/T3c	19 (3.4)/ 17 (3.0)/14 (2.5)	
N 0/1	514 (92.1)/44 (7.9)	
M 0/1	520 (93.2)/38 (6.8)	
R.E.N.A.L. score		100
Low (4–6)	225 (40.3)	
Intermediate (7–9)	286 (51.3)	
High (10–12)	47 (8.4)	
Tumor maximal diameter, cm	5.5 (3.2–7.8)	100
Operation time, min	130 (100–170)	100
Bleeding and transfusion amount		
pRBC transfusion, *n*	52 (9.3)	100
Estimated blood loss, mL	200 (100–400)	99.6
Anesthesia-related parameters		
Volatile anesthetics use, *n*	494 (88.5)	
Total intravenous anesthesia, *n*	64 (11.5)	
Crystalloid administration, mL	1100 (750–1500)	100
Colloid administration, mL	0 (0–300)	100
Vasopressor infusion during surgery	29 (5.2)	100

Data are presented as median (interquartile range) or number (%). AKI = acute kidney injury; eGFR = estimated glomerular filtration rate; R.E.N.A.L. = radius, exophytic/endophytic properties, nearness of tumor to collecting system or sinus, anterior/posterior, hilar, location relative to polar lines; pRBC = packed red blood cell.

**Table 2:** Cox proportional hazard regression analysis for new-onset chronic kidney disease or all-cause mortality during the three years after radical nephrectomy in all patients (*n* = 558).

Variable	Hazard Ratio	95% CI	*p*-Value
Age, years	1.05	1.00–1.09	0.043
Female	1.30	0.81–2.10	0.368
Body-mass index, kg/m^2^	1.01	0.95–1.08	0.769
History of hypertension	1.70	1.07–2.78	0.022
History of diabetes mellitus	1.95	1.13–3.44	0.012
Preoperative hemoglobin, g/dL	1.14	0.99–1.30	0.064
Preoperative albumin, g/dL	0.63	0.33–1.12	0.077
Preoperative proteinuria, *n*	0.82	0.42–1.80	0.547
Preoperative hematuria, *n*	1.12	0.57–1.74	0.657
Preoperative estimated glomerular filtration rate, mL/min/1.73m^2^	0.99	0.98–0.99	0.042
Postoperative acute kidney injury	2.46	1.70–3.63	<0.001
No acute kidney injury	baseline		
Acute kidney injury stage 1	1.71	1.25–2.32	<0.001
Acute kidney injury stage 2 or 3	2.72	1.78–4.10	<0.001
Preoperative tumor maximal diameter, cm	1.05	0.97–1.12	0.164
Open surgery (vs. laparoscopic or robot-assisted)	0.74	0.49–1.15	0.255
Operation time, hour	0.96	0.81–1.18	0.847
Total intravenous anesthesia	0.89	0.61–1.35	0.558
Intraoperative crystalloid administration, per 100 mL	0.87	0.62–1.28	0.415
Intraoperative colloid administration, per 100 mL	1.06	0.98–1.16	0.176
Intraoperative vasopressor infusion, *n*	0.94	0.92–1.17	0.514
Red blood cell transfusion, *n*	0.82	0.37–1.75	0.427

CI = confidence interval. Intraoperative vasopressor infusion means norepinephrine or phenylephrine infusion during surgery.

**Table 3:** Multivariable linear regression analysis of functional change ratio after radical nephrectomy.

Variable	β ± Standard Error	*p*-Value	VIF
Age, years	0.012 ± 0.001	0.057	1.69
Female	0.037 ± 0.031	0.240	1.30
Body-mass index, kg/m^2^	0.002 ± 0.005	0.647	1.14
History of hypertension	−0.030 ± 0.011	0.047	1.52
History of diabetes mellitus	−0.044 ± 0.018	0.044	1.24
Preoperative hemoglobin concentration, g/dL	0.007 ± 0.010	0.411	2.06
Preoperative albumin level, mg/dL	0.033 ± 0.041	0.481	1.82
Preoperative proteinuria	−0.034 ± 0.047	0.470	1.45
Preoperative estimated glomerular filtration rate, per 10 mL/min/1.73 m^2^	0.170 ± 0.122	0.002	1.26
Postoperative acute kidney injury	−0.168 ± 0.322	0.011	1.16
Maximal diameter of renal mass, cm	0.002 ± 0.004	0.572	1.28
Open surgery (vs. laparoscopic or robot-assisted)	−0.030 ± 0.028	0.228	1.19
Operation time, hour	−0.016 ± 0.012	0.179	1.20
Total intravenous anesthesia	0.069 ± 0.056	0.375	1.10
Intraoperative crystalloid administration, mL/kg	−0.011 ± 0.023	0.724	1.45
Intraoperative colloid administration, mL/kg	−0.001 ± 0.004	0.717	1.37
Intraoperative red cell transfusion, *n*	0.096 ± 0.049	0.150	1.39

VIF = variance inflation factor. The functional change ratio was determined as a ratio of the most recent estimated glomerular filtration rate (eGFR) (at least 24 months and up to three years after surgery) to eGFR at 3 to 12 months.

## Supplementary Materials

The following is available online https://www.mdpi.com/2077-0383/9/3/619/s1. Figure S1. Kaplan–Meier survival curve analysis for new-onset chronic kidney disease stage 3a or higher (estimated glomerular filtration rate < 60 mL/min/1.73 m^2^) or all-cause mortality as the primary outcome between different type of surgical procedure groups; Table S1. The number of patients who had follow-up values of renal function; Table S2. Comparison of the incidence of acute kidney injury between the types of surgical procedures; Table S3. Comparison of the incidence of complications between the patients with and without acute kidney injury according to Clavien-Dindo classification.
