# Supplementary material for: Robust Association between Acute Kidney Injury after Radical Nephrectomy and Long-term Renal Function
Source: J Clin Med. 2020 Feb 25;9(3):619. doi: 10.3390/jcm9030619 (PMC7141119; doi:10.3390/jcm9030619)
Supplement: Supplementary file 1 [file jcm-09-00619-s001.pdf]

Supplementary Materials

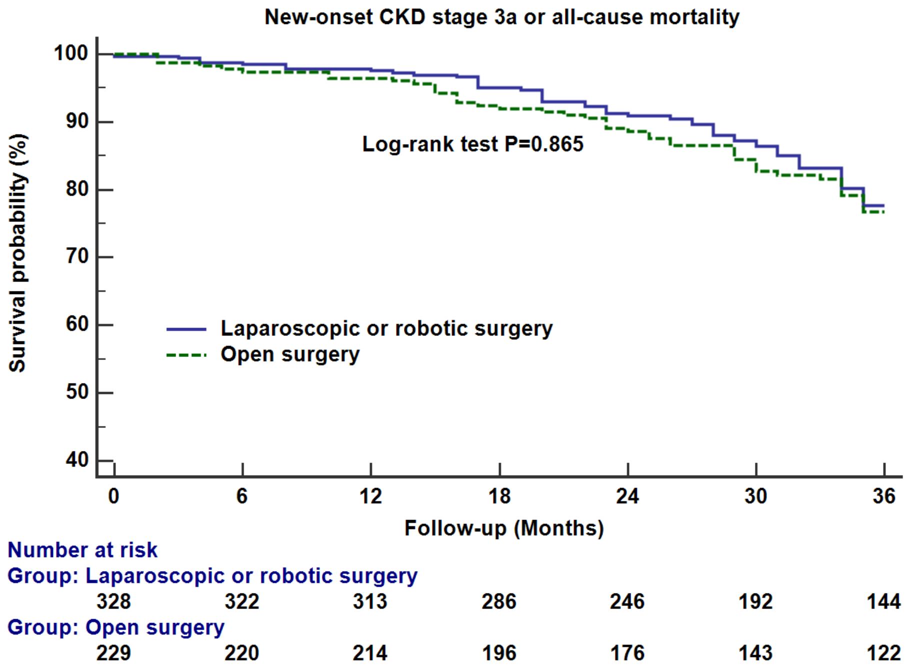

Figure S1. Kaplan-Meier survival curve analysis for new-onset chronic kidney disease stage 3a or higher (estimated glomerular filtration rate <60 ml/min/1.73m^2) or all-cause mortality as the primary outcome between different type of surgical procedure groups. The result of Log-rank test is shown on the figure. The numbers of patients who had follow-up eGFR values at each time point were shown in the bottom of the figure.

Table S1. The number of patients who had follow-up values of renal function.

	6 months	12 months	18 months	24 months	30 months	36 months
Follow-up	542 (97.1)	527 (94.4)	482 (86.4)	422 (75.6)	335 (60.0)	261 (46.8)
Lost to follow-up	16 (2.9)	31 (5.6)	76 (13.6)	136 (24.4)	223 (40.0)	297 (53.2)

Data are presented as number (%).

Table S2. Comparison of the incidence of acute kidney injury between the types of surgical procedures. .

	Open surgery (n=329)	Laparoscopic or robotic surgery (n=229)	P-value
No AKI	164 (49.8)	153 (66.8)	
AKI stage 1	103 (31.3)	57 (24.9)	<0.001*
AKI stage 2 or 3	62 (18.8)	19 (8.3)	
AKI all stage	165 (50.2)	76 (33.2)	<0.001

Data are presented as number (%). AKI = acute kidney injury. P-values are the results of the chi-square test. * Comparing the incidence of no AKI, AKI stage 1, and stage 2 or 3 between surgical procedure groups.

Table S3. Comparison of the incidence of complications between the patients with and without acute kidney injury according to Clavien-Dindo classification.

	No AKI group (n=317)	AKI group (n=241)	P-value
Grade I	4 (1.3)	8 (3.3)	0.104
Grade II	3 (0.9)	1 (0.4)	
Grade IIIa	3 (0.9)	1 (0.4)	
Grade IIIb	2 (0.6)	-	
Grade IVa	1 (0.3)	2 (0.8)	
Grade IVb	-	-	
Grade V	-	-	

Data are presented as number (%). AKI = acute kidney injury. P-values are the result of the chi-square test between no-AKI and AKI groups.
